# Social Cognition 2.0: Toward Mechanistic Theorizing

**DOI:** 10.3389/fpsyg.2019.02643

**Published:** 2019-11-29

**Authors:** Diana Kim, Bernhard Hommel

**Affiliations:** ^1^Institute for Psychological Research, Leiden University, Leiden, Netherlands; ^2^Leiden Institute for Brain and Cognition, Leiden University, Leiden, Netherlands

**Keywords:** conformity, theory of event coding, adaptive behavior, social cognition, mechanistic theorizing

## Abstract

Social cognition emerged in the 1970s and 80s as an attempt to answer social-psychological questions by adopting experimental techniques and theoretical concepts from cognitive psychology. Recently, cognitive psychologists began to build complementary bridges between cognitive and social psychology by showing increasing interest in the cognitive implications of social situations. Here, we take a closer look at the remaining obstacles to join cognitive and social perspectives on human behavior. Using conformity as an example, we attempt to demonstrate that the social-cognition approach has been successful in adopting cognitive concepts and experimental methods, but is still lagging behind with respect to (1) mechanistic theorizing, as it often engages in merely describing phenomena in terms of reasons rather than explaining it in terms of causes and (2) reflecting the sociohistorical context of the phenomenon under investigation. As we try to show, developing mechanistic theories for social phenomena, including the effects of individual differences and their sociohistorical dependencies, is not only possible but necessary to eliminate the boundaries between cognitive and social accounts of human behavior.

Social cognition, a “field of psychology concerned with the mental processes through which we preceive, think about, and act toward other people and in response to situational factors” (Amodio, 2019), emerged in the 1970s and 80s, as a result of adopting experimental techniques and theoretical concepts from cognitive psychology. In contrast to more sociological or behavioristic approaches, the social cognition approach tries to understand social thought and behavior from an individualistic perspective that considers the way information about social events is processed, stored, and used. This emphasis on individuals has been criticized (e.g., Taylor and Brown, 1979), and we do not intend to claim that the social cognition is the best or only way to understand social behavior. And yet, the social-cognition approach does provide an interesting and stimulating interface between cognitive and social research and theorizing, which is particularly important as the increasing interest of social psychologists in cognitive processes has recently been echoed by an increasing interest of cognitive psychologists in social situations and the cognitive implications thereof (Hommel, 2006).

This interest was fueled by surprising observations that what cognitive psychologists considered well-established, almost hard-wired cognitive effects can be strongly affected by the real or even imagined presence of other people. Take the notorious Simon effect (Simon, 1969), which indicates that speeded responses to stimuli are faster and more accurate if the stimulus spatially corresponds to the location of the response, even if stimulus location is irrelevant to the task. The classic effect has been replicated hundreds, perhaps thousands of times across different variations of the basic design, the stimuli, and the responses (e.g., Hommel, 2011). It is commonly attributed to response conflict, the idea being that, when stimulus and response do not correspond, the intentional response conflicts with the response that is automatically triggered by the stimulus location, which in turn may lead to an error or a delay that reflects resolution of the conflict (e.g., Kornblum et al., 1990). If so, one would expect that the effect disappears if the participant responds with only one key to only one of the stimuli, rendering the task a “go-nogo task,” because in this case the correct response can already be prepared long before the stimulus appears. This is indeed what studies have shown (Hommel, 1996). However, if such a go-nogo version of the task is performed in the presence of another person who operates the other key to respond to the other stimulus (see Figure 1), the effect comes back—the so-called Joint Simon effect (JSE; Sebanz et al., 2003).

**Figure 1:**
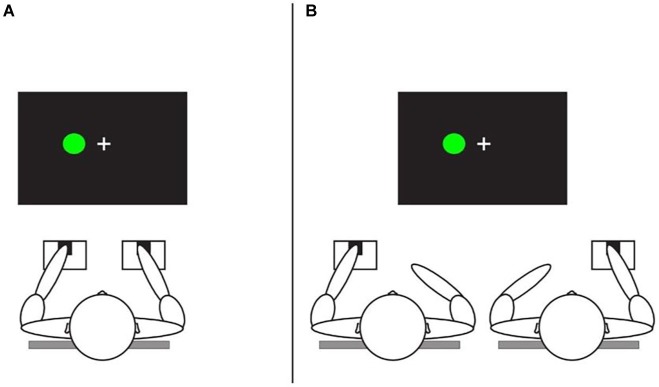
Schematic representation of standard Simon task **(A)** and joint Simon tasks **(B)** (Ruissen and de Bruijn, 2016).

The discovery of the JSE has been considered to show that people automatically co-represent other people and their tasks and to demonstrate “the fundamental social nature of perception and action” (Knoblich and Sebanz, 2006). While more recent findings have challenged the assumption of automatic co-representation (Dolk et al., 2014) and speculations about “social nature” do not seem to contribute much to understanding the underlying mechanisms, the observation of the JSE and related phenomena demonstrate that even the most basic cognitive effects are not immune to the social environment and the real or imagined presence of others. This in turn renders any clear-cut separation between cognitive and social psychology or phenomena questionable (Hommel, 2006)—irrespective of whether one assumes that all social behavior is actually cognitive in nature or whether one prefers the opposite perspective. However, the increasing methodological overlap between the cognitive and social sciences notwithstanding, integrating insights from both fields still faces considerable challenges. In the following, we highlight two of them: non-mechanistic theorizing about social effects and insufficient attention for the historicity and cultural boundedness of social phenomena. We will discuss both issues in the context of social conformity, by drawing on some, partly rather unexpected recent findings from our lab.

## Non-Mechanistic Theorizing

Conforming behavior has always been an interesting social phenomenon attracting the attention of many social psychologists in all times. In his most famous, but at the same time most controversial study, Asch (1951) demonstrated that people easily change their opinion when confronted with deviating opinions of others. His participants were to choose the one out of three visual lines that would match a reference line in length. Even though the correct response was obvious, participants were strongly affected by deviating judgments of other participants, who in fact were all confederates, and followed the group’s opinion even when it was incorrect. Asch himself initially expected participants to choose the correct response irrespective of the group’s opinion, so the actual results surprised him as much as the entire scientific community at that time.

How can we explain this kind of behavior? Social-psychological accounts have claimed that conformity effects reflect the belief in the superior knowledge of the group (Asch, 1951) and suggested that people conform to the group’s norms and values because they want to be accepted by it (e.g., Kelman, 1958). While we do not doubt the validity of these claims, they remain descriptive and do not amount to a truly mechanistic explanation. Social-psychological “explanations” refer to the possible reasons of conformity behavior rather than to the actual causes, and thus favor the personal level of explanation over the more appropriate functional or systems level. If we are to understand social behavior by referring to and analyzing the information-processing operations underlying it, explanations should be restricted to this functional/systems level of description rather than to the personal level at which the to-be-explained phenomenon is defined. Failing to do so is likely to result in re-describing the phenomenon in pseudo-explanatory terminology, rather than revealing the actual mechanistic underpinnings (Hommel, 2019). Neuroscientific “explanations,” in turn, have merely demonstrated that conformity behavior goes along with neural activity that has also been shown to go along with other (e.g., conflict-inducing) behavior that is similar to conformity behavior. For example, in the study of Klucharev et al. (2009), results demonstrated that when individual judgments differed from those of the group, activity in the medial prefrontal cortex (an area generally known to be involved in the processing of conflict) increased, while activity in the nucleus accumbens, an area associated with the expectation of reward, decreased. Again, we do not doubt the validity and importance of these observations, but they hardly provide a mechanistic principle that would really explain the phenomenon.

We conclude that the social cognition approach was successful in bringing into play cognitive methodological techniques and cognitive theoretical concepts, but it did not yet make the decisive step of engaging in truly mechanistic theorizing. In order to do so, social cognition approaches would not only need to include cognitive concepts in the explanation of phenomena but also to delineate exactly how the interaction of cognitive operations causally generates the phenomena. A truly mechanistic approach needs to look for a cognitive theory that goes beyond re-describing or provide personal reasons to produce effects like conformity by providing a mechanism that allows researchers to systematically predict such behavior under various conditions.

To become more explicit with respect to the needed level theorizing and to provide a concrete example case, we refer to a recent attempt of ours (Kim and Hommel, 2015) to explain key characteristics of conformity by using basic principles of the Theory of Event Coding (TEC), a general theory of the interactions between human perception and action (Hommel et al., 2001). We would like to emphasize that our goal is not to defend our particular approach at this point, but will use it only as an example for the degree of theoretical detail and specificity that we think is needed for the next generation of social cognition approaches—Social Cognition 2.0 (a term we borrow from Amodio, 2019) that is. We also admit that our approach does not account for all available conformity-related observations. In the original Asch (1951) experiments, participants carried out their judgments in the presence of confederates. This introduces motives of justification and self-presentation, as obvious from self-reports showing that some participants knew that the answer of the group was wrong but they apparently did not dare to give a deviant answer. Later, approaches have focused more on the after-effects of information about majority opinions and judgments of a relevant reference group, such as in the studies described below (i.e., changes of one’s judgment of a given object or issue to make it more compatible with a majority vote), and it is only these kinds of after-effects we will be addressing in the following.

Our account of the impact of majority judgments on the future behavior of participants was motivated by TECs claim that both produced and perceived events (i.e., action plans and perceptual representations) are coded in terms of their features and in a common format (Hommel et al., 2001; Hommel, 2009), and that it does not differentiate between representing “me/self” and “others” (Hommel, 2018). Accordingly, actions performed by the individual him/herself and the actions performed by someone else should be coded in comparable ways (Hommel et al., 2009)—even though one commonly has more (e.g., proprioceptive, anticipatory, and historical) information about one’s own action (Hommel, 2018). Given TECs further assumption that co-activated event codes tend to be bound into event files (Hommel, 2004), experiencing a (self- or other-performed) action should lead to a binding of action-related feature codes to codes representing the perceptual context (i.e., the stimuli inducing the action, the object on which it is carried out, and the situation in which that happens). Event files operate according to a pattern-completion logic, so that the retrieval or stimulus-induced reactivation of one code spreads to the other components (Kühn et al., 2011).

Given these assumptions, we reasoned, conformity-related changes in judgment might thus have nothing to do with group wisdom or group norms but rather reflect a mix-up of event files reflecting one’s own action history and event files reflecting the actions of others. If, say, an individual reacts to a stimulus line by judging it to be 2 cm long, she would store an event file that integrates perceptual codes of the stimulus features and the response “2 cm.” Perceiving nine other people reacting to the same stimulus by judging it to be 3 cm long would lead to the storage of nine event files that integrate stimulus features with the response “3 cm.” Even if event files coding one’s own action might enjoy more attention or stronger weighting, encountering the stimulus on another occasion would still tend to retrieve 10 event files with most of them suggesting another than the correct response. Given the notorious strong impact of (memories of) past responses on present performance (Lewin, 1922a,b), what looks like conformity may simply be the failure to properly discriminate between one’s own (correct) response and the (incorrect) response(s) of one’s co-actor(s).

To test this possibility, Kim and Hommel (2015) adopted the experimental design of recent conformity studies (Klucharev et al., 2009; Shestakova et al., 2012) and had participants rate 220 pictures of female faces for attractiveness on a scale 1–8. After their own evaluation, participants were presented with what they were led to believe was the rating of the same face by other students of their university—an important reference group. After a short break, participants were asked to rate the same faces again. Replicating previous studies, we found significant changes in the ratings into the direction of the rating of a reference group: higher scores if a reference group found the face more attractive and lower scores if they found it less attractive. Importantly, we included a second group of participants that was also presented with other ratings after having rated each face themselves, but the cover story did not mention any possible reference group. Rather, participants were told that the numbers were randomly chosen and function as distractors to make the task more difficult. Given our theoretical background, we expected that the mere exposure to an alternative “response” (even in the absence of any social indication of it) would result in an adjustment of participants’ rating behavior in the second rating session. Indeed, even in the absence of any social cover story, participants demonstrated “conformity” by adjusting their second rating into the direction of the presented number (Kim and Hommel, 2015).

We hasten to add that this observation must not be taken as unequivocal evidence for the validity of our theoretical background. As argued by Ihmels and Ache (2018) and partly confirmed by Kim and Hommel (2018), the paradigm used by Kim and Hommel (2015) and previous researchers is sensitive to regression-to-the-mean effects, which implies that the effect that Kim and Hommel (2015) were able to extend to non-social conditions was actually not a real conformity effect. What we do want to emphasize, however, is that we consider our approach the first truly mechanistic account of conformity behavior which allows for much more specific predictions than the previously suggested descriptive accounts.

## Ahistorical Theorizing

A major objection against the application of cognitive theories to social phenomena may be based on the fact that cognitive approaches address processes that rely on millions of years of biological evolution while social phenomena can change even within the lifespan of one generation. This problem was voiced by Gergen (1973), who argued that laws and principles of social interaction vary over time, so that “theories of social behavior are primarily reflections of contemporary history”. If so, phenomena we observe today may become weaker tomorrow and even cease to exist the year after, sometimes due to that reporting and discussing a phenomenon might work back and speed up eliminating the phenomenon; e.g., studying and discussing obedience to authority may reduce the likelihood of finding obedience in citizens.

A related aspect of social behavior is its cultural variability. Similar social situations can evoke different behavioral responses in members of different cultures, historical traditions, and religions, different gender roles, even different emotional reactions, and different neural responses (Han and Ma, 2014) to life events like death or childbirth. This suggests that culture has a strong impact on basic cognitive aspects of human brain functioning. Indeed, a number of seminal studies by Nisbett and colleagues, who have strongly revived the interest in cultural factors, found that people growing up in East Asian cultures such as China and Japan tend to develop more holistic thinking styles, whereas people brought up in Western cultures like Australia and the USA tend to develop analytic thinking styles, with severe consequences for the way global and local information is processed (e.g., Nisbett et al., 2001). Along the same lines, members of collectivistic (e.g., Catholicism or Buddhism) and individualistic (e.g., neo-Calvinism) religions show profound differences in cognitive-control styles (Hommel and Colzato, 2017).

Take again conformity as an example. After Asch’s famous experiment (Asch, 1951), many replications of the study followed exploring which factors contributed to the main effect, such as the obviousness of the correct response, group size, or gender composition of the group (e.g., Larsen, 1990; Bond, 2005). But not all replications were successful. In fact, effect sizes decreased systematically over decades and even tended to disappear in the most recent studies (Bond and Smith, 1996), which according to Perrin and Spencer (1981) suggests that Asch’s study was a “child of its time” by reflecting the high level of social obedience of 1950s in the USA that disappeared as a consequence of the rise of individualistic values. And yet, successful replications of the Asch’s observations are still reported even in the Western World in the last two decades of the 20th century (e.g., Vlaander and Rooijen, 1985; Abrams et al., 1990). Could this be a sign that Gergen was correct in claiming that social phenomena cannot be studied in the lab?

We would like to argue that, on the contrary, Gergen’s challenge calls for more mechanistic theorizing based on systematic laboratory research. Historical processes and the dynamics of social phenomena they imply do not stand in the way of more rigorous theorizing but rather provide useful constraints for building theories that embrace historical processes and make them part of the modeling process. Hence, we need mechanistic models that can explain exactly how historical changes impact cognitive processing and the phenomenon under investigation. For example, Hommel and Colzato (2017) have suggested that differential emphasis on commonalities versus distinctions between events and individuals in collectivistic and individualistic cultures provide selective social feedback for the development of a more integrative or a more discriminative cognitive processing style, which eventually establishes a corresponding default bias. A more integrative bias would increase, and a more focused bias would reduce the amount of social information considered in a decision, which in the latter case would reduce and eventually eliminate phenomena that rely on the processing of social information, such as conformity. If so, conformity effects should be easier to find in more collectivistic societies.

**Figure 2:**
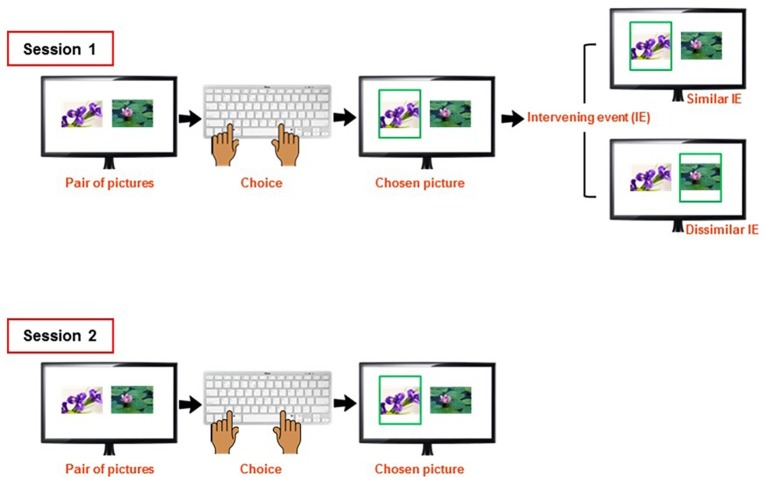
Schematic representation of the sequence of events occurring on each trial of the conformity task of Study 2.

To test this prediction, Kim et al. (2019, submitted) created a novel paradigm for testing conformity effects that avoids methodological problems like regression-to-the-mean effects. Participants saw 110 pairs of pictures (plants and flowers) and chose the one they liked more. As in previous studies, participants were then confronted with the opinion of “others” before they were tested again to see whether deviating opinions of “others” changed their judgment (see Figure 2). To compare collectivistic and individualistic cultures, we tested participants in the Netherlands, a country in the top-5 Hofstede’s Individualism/Collectivism Scale (Hofstede et al., 2010, with 80 out of 100 Individualism points), and China (20 Individualism points), which despite a visible trend toward individualization has remained much more collectivistic in comparison (Van de Vliert et al., 2013). We found a significant main effect of conformity in the Chinese but not in the Dutch sample, suggesting that the Dutch students were ignoring the social information. This suggests that conformity behavior is not hardwired but emerges from an interaction between cultural biases and situational salience of social information. Rigorous mechanistic theorizing can capture both of these factors and make specific and increasingly precise predictions that are open to laboratory testing.

## Conclusions

Lewin (1952) claimed that nothing is as practical as a good theory, and we claim that this holds in particular for the understanding of social phenomena. While the social-cognition approach has been successful in adopting cognitive concepts and experimental methods, it is still lagging behind with respect to mechanistic theorizing and still way too often engages in merely describing phenomena in terms of reasons rather than explaining it in terms of causes. As we tried to show here, developing mechanistic theories for social phenomena is possible (see also Pfister et al., 2019, for a mechanistic account of rule-breaking), and there is no reason to shy away from using these theories to also account for the impact of historical development, cultural and individual variability, and environmental dynamics. In other words, the time is ripe for Social Cognition 2.0.

## Author Contributions

Both authors contributed to the development of the article concept and they both approved the final version of the manuscript. DK drafted the manuscript and BH provided critical revisions.

### Conflict of Interest

The author declares that the research was conducted in the absence of any commercial or financial relationships that could be construed as a potential conflict of interest.

The reviewer RP declared a past co-authorship with one of the authors BH to the handling editor.

## References

[ref1] Abrams D. Wetherell M. Cochrane S. Hogg M. A. Turner J. C. (1990). Knowing what to think by knowing who you are: self categorisation and the nature of norm formation, conformity and group polarisation. Br. J. Soc. Psychol. 29, 97–119. 10.1111/j.2044-8309.1990.tb00892.x, PMID: 2372667

[ref2] Amodio M. D. (2019). Social cognition 2.0: an interactive memory. Trends Cogn. Sci. 23, 21–33. 10.1016/j.tics.2018.10.002, PMID: 30466793

[ref3] Asch S. E. (1951). “Effects of group pressure upon the modification and distortion of judgments” in Groups, leadership, and men. ed. Guetzkow H. (Pittsburgh: Carnegie Press), 177–190.

[ref4] Bond R. (2005). Group size and conformity. Group Process. Intergroup Relat. 8, 331–354. 10.1177/1368430205056464

[ref5] Bond R. Smith P. B. (1996). Culture and conformity: a meta-analysis of studies using Asch’s (1952b, 1956) line judgment task. Psychol. Bull. 119, 111–137. 10.1037/0033-2909.119.1.111

[ref7] Dolk T. Hommel B. Colzato L. Schütz-Bosbach S. Prinz W. Liepelt R. (2014). The joint Simon effect: a review and theoretical integration. Front. Psychol. 5:974. 10.3389/fpsyg.2014.00974, PMID: 25249991 PMC4155780

[ref8] Gergen K. J. (1973). Social psychology as history. J. Pers. Soc. Psychol. 26, 309–320. 10.1037/h0034436

[ref9] Han S. Ma Y. (2014). Cultural differences in human brain activity: a quantitative meta-analysis. NeuroImage 99, 293–300. 10.1016/j.neuroimage.2014.05.062, PMID: 24882220

[ref10] Hofstede G. Hofstede G. J. Minkov M. (2010). *Cultures and organizations: Software of the mind*. 3rd Edn. London, UK: McGraw-Hill.

[ref500] Hommel B. (1996). S-R compatibility effects without response uncertainty. Q. J. Exp. Psychol. 49A, 546–571. 10.1080/027249896392496, PMID: 15491903

[ref11] Hommel B. (2004). Event files: feature binding in and across perception and action. Trends Cogn. Sci. 8, 494–500. 10.1016/j.tics.2004.08.007, PMID: 15491903

[ref12] Hommel B. (2006). “Bridging social and cognitive psychology?” in Bridging social psychology: The benefits of transdisciplinary approaches. ed. Van Lange P. A. M. (Hillsdale, NJ: Erlbaum), 167–172.

[ref13] Hommel B. (2009). Action control according to TEC (theory of event coding). Psychol. Res. 73, 512–526. 10.1007/s00426-009-0234-2, PMID: 19337749 PMC2694931

[ref14] Hommel B. (2011). The Simon effect as tool and heuristic. Acta Psychol. 136, 189–202. 10.1016/j.actpsy.2010.04.011, PMID: 20507830

[ref15] Hommel B. (2018). Representing oneself and others: an event-coding approach. Exp. Psychol. 65, 323–331. 10.1027/1618-3169/a000433, PMID: 30638165 PMC6716141

[ref501] Hommel B. (2019). Pseudo-mechanistic explanations in psychology and cognitive neuroscience. Top. Cogn. Sci. 10.1111/tops.12448 [Epub ahead of print]., PMID: 31359621 PMC7687254

[ref16] Hommel B. Colzato L. S. (2017). The social transmission of metacontrol policies: mechanisms underlying the interpersonal transfer of persistence and flexibility. Neurosci. Biobehav. Rev. 81, 43–58. 10.1016/j.neubiorev.2017.01.009, PMID: 28088534

[ref17] Hommel B. Colzato L. S. van den Wildenberg W. P. M. (2009). How social are task representations? Psychol. Sci. 20, 794–798. 10.1111/j.1467-9280.2009.02367.x, PMID: 19493327

[ref18] Hommel B. Müsseler J. Aschersleben G. Prinz W. (2001). The theory of event coding (TEC): a framework for perception and action planning. Behav. Brain Sci. 24, 849–878. 10.1017/S0140525X01000103, PMID: 12239891

[ref19] Ihmels M. Ache F. (2018). Event based conformity vs. regression to the mean: a comment on Kim and Hommel. Psychol. Sci. 29, 1190–1192. 10.1177/0956797617719082, PMID: 29461927

[ref20] Kelman H. C. (1958). Compliance, identification, and internalization: three processes of attitude change. J. Confl. Resolut. 2, 51–60.

[ref21] Kim D. Hommel B. (2015). An event-based account of conformity. Psychol. Sci. 26, 484–489. 10.1177/0956797614568319, PMID: 25749696

[ref22] Kim D. Hommel B. (2018). Reply to Ihmels and Ache (2018): event-based conformity versus regression to the mean. Psychol. Sci. 29, 1193–1194. 10.1177/0956797618773095, PMID: 29738284

[ref23] Klucharev V. Hytönen K. Rijpkema M. Smidts A. Fernández G. (2009). Reinforcement learning signal predicts social conformity. Neuron 61, 140–151. 10.1016/j.neuron.2008.11.027, PMID: 19146819

[ref24] Knoblich G. Sebanz N. (2006). The social nature of perception and action. Curr. Dir. Psychol. Sci. 15, 99–104. 10.1111/j.0963-7214.2006.00415.x

[ref25] Kornblum S. Hasbroucq T. Osman A. (1990). Dimensional overlap: cognitive basis for stimulus-response compatibility - a model and taxonomy. Psychol. Rev. 97, 253–270. 10.1037/0033-295X.97.2.253, PMID: 2186425

[ref26] Kühn S. Keizer A. Colzato L. S. Rombouts S. A. R. B. Hommel B. (2011). The neural underpinnings of event-file management: evidence for stimulus-induced activation of, and competition among stimulus-response bindings. J. Cogn. Neurosci. 23, 896–904. 10.1162/jocn.2010.21485, PMID: 20377359

[ref27] Larsen K. S. (1990). The Asch conformity experiment: replication and transhistorical comparisons. J. Soc. Behav. Pers. 5, 163–168.

[ref28] Lewin K. (1922a). Das Problem der Willensmessung und das Grundgesetz der Assoziation I. Psychol. Forsch. 1, 191–302.

[ref29] Lewin K. (1922b). Das Problem der Willensmessung und das Grundgesetz der Assoziation II. Psychol. Forsch. 2, 65–140.

[ref30] Lewin K. (1952). Field theory in social science: Selected theoretical papers by Kurt Lewin. London: Tavistock.

[ref31] Nisbett R. Peng K. Choi I. Norenzayan A. (2001). Culture and systems of thought: holistic vs. analytic cognition. Psychol. Rev. 108, 291–310. 10.1037/0033-295X.108.2.291, PMID: 11381831

[ref32] Perrin S. Spencer C. P. (1981). Independence or conformity in the Asch experiment as a reflection of cultural and situational factors. Br. J. Soc. Psychol. 20, 205–210. 10.1111/j.2044-8309.1981.tb00533.x

[ref33] Pfister R. Wirth R. Weller L. Foerster A. Schwarz K. A. (2019). Taking shortcuts: cognitive conflict during motivated rule-breaking. J. Econ. Psychol. 71, 138–147. 10.1016/j.joep.2018.06.005

[ref100] Ruissen M. I. de Bruijn E. R. A. (2016). Competitive game play attenuates self-other integration during joint task performance. Front. Psychol. 7:274. 10.3389/fpsyg.2016.00274 26973571 PMC4776308

[ref34] Sebanz N. Knoblich G. Prinz W. (2003). Representing others‘actions: just like one’s own? Cognition 88, B11–B21. 10.1016/S0010-0277(03)00043-X, PMID: 12804818

[ref35] Shestakova A. Rieskamp J. Tugin S. Ossadtchi A. Krutitskaya J. Klucharev V. (2012). Electrophysiological precursors of social conformity. Soc. Cogn. Affect. Neurosci. 8, 756–763. 10.1093/scan/nss064 22683703 PMC3791064

[ref36] Simon J. R. (1969). Reactions toward the source of stimulation. J. Exp. Psychol. 81, 174–176. 10.1037/h0027448, PMID: 5812172

[ref38] Taylor D. M. Brown R. J. (1979). Towards a more social social psychology? Br. J. Soc. Clin. Psychol. 18, 173–180. 10.1111/j.2044-8260.1979.tb00322.x

[ref6] Van de Vliert E. Yang H. Wang Y. Ren X. (2013). Climato-economic imprints on Chinese collectivism. J. Cross Cult. Psychol. 44, 589–605. 10.1177/0022022112463605

[ref39] Vlaander G. van Rooijen L. (1985). Independence and conformity in Holland: Asch’s experiment in Holland three decades later. Gedrag: Tijdschrift voor Psychologie 13, 49–55.

