# Corrigendum: Social Cognition 2.0: Toward Mechanistic Theorizing

**DOI:** 10.3389/fpsyg.2020.00041

**Published:** 2020-02-05

**Authors:** Diana Kim, Bernhard Hommel

**Affiliations:** ^1^Institute for Psychological Research, Leiden University, Leiden, Netherlands; ^2^Leiden Institute for Brain and Cognition, Leiden University, Leiden, Netherlands

**Keywords:** conformity, theory of event coding, adaptive behavior, social cognition, mechanistic theorizing

In the original article, there was a mistake in the legend for **Figure 1**. Schematic representation of standard Simon task **(A)** and joint Simon tasks **(B)**. The figure was originally created by (Ruissen and de Bruijn, 2016). The correct legend appears below.

**Figure 1**. Schematic representation of standard Simon task **(A)** and joint Simon tasks **(B)**. (Ruissen and de Bruijn, 2016).

The authors apologize for this error and state that this does not change the scientific conclusions of the article in any way. The original article has been updated.
